# malERA: An updated research agenda for insecticide and drug resistance in malaria elimination and eradication

**DOI:** 10.1371/journal.pmed.1002450

**Published:** 2017-11-30

**Authors:** The malERA Refresh Consultative Panel on Insecticide and Drug Resistance

## Abstract

Resistance to first-line treatments for *Plasmodium falciparum* malaria and the insecticides used for *Anopheles* vector control are threatening malaria elimination efforts. Suboptimal responses to drugs and insecticides are both spreading geographically and emerging independently and are being seen at increasing intensities. Whilst resistance is unavoidable, its effects can be mitigated through resistance management practices, such as exposing the parasite or vector to more than one selective agent. Resistance contributed to the failure of the 20th century Global Malaria Eradication Programme, and yet the global response to this issue continues to be slow and poorly coordinated—too often, too little, too late. The Malaria Eradication Research Agenda (malERA) Refresh process convened a panel on resistance of both insecticides and antimalarial drugs. This paper outlines developments in the field over the past 5 years, highlights gaps in knowledge, and proposes a research agenda focused on managing resistance. A deeper understanding of the complex biological processes involved and how resistance is selected is needed, together with evidence of its public health impact. Resistance management will require improved use of entomological and parasitological data in decision making, and optimisation of the useful life of new and existing products through careful implementation, combination, and evaluation. A proactive, collaborative approach is needed from basic science and the development of new tools to programme and policy interventions that will ensure that the armamentarium of drugs and insecticides is sufficient to deal with the challenges of malaria control and its elimination.

## Abstract 2

Janet Hemingway and colleagues examine progress in research on insecticide and drug resistance in malaria elimination and eradication and propose a research agenda.

**Summary points**

- Since 2011, significant progress has been made in understanding resistance. Surveillance has been expanded and improved in many malaria-endemic countries and there is a better understanding of the genetic basis of resistance, identifying some molecular markers that can be used to track its emergence and spread. Better tools to measure and manage the intensity of resistance are available.
- However, our response to increases in the prevalence and intensity of resistance has been slow and reactive. A promising pipeline of new vector control tools and therapeutics is in development, but all actors in the malaria community need to plan proactively how to implement, integrate, and evaluate these products.
- Quantifying the public health impact of resistance has been difficult, particularly for insecticides. For both insecticides and drugs, defining the minimum essential evidence required for policy makers to manage resistance and ensuring that programs employ rigorous quality assurance in collecting and managing these data are critical.
- As malaria control increases, the selection pressure on the parasite or mosquito vector increases. Strategies for resistance management are therefore crucial for all stages of elimination. Countries need to allocate funding and human resources to effectively manage the threat of resistance and sustain the gains achieved to date.
- This paper reviews the current knowledge base and identifies research priorities addressing resistance to drugs and insecticides. It is a result of a unique collaborative effort of experts in drug and insecticide resistance brought together for the malERA Refresh process.

## Introduction and rationale

Over the past decade, unprecedented progress has been made in reducing malaria morbidity and mortality [1]. However, growing resistance to the first-line treatment for *P*. *falciparum* malaria, artemisinin-based combination therapies (ACTs), and the insecticides used to suppress mosquito vectors threaten the sustainability of recent gains in malaria control and longer-term prospects for elimination.

Vector control and antimalarial treatment depend on a limited armamentarium, and when single drugs and insecticides are widely deployed, selection pressure is intense and the emergence of resistant parasites and mosquitoes is inevitable.

Drug and insecticide resistance were crosscutting issues in the original malERA (Malaria Eradication Research Agenda) series in 2011 [2]. However, the parasite and vector communities rarely interact. The increasing urgency of these issues and the contrasting operational responses warranted a dedicated panel in the malERA Refresh process. The failure of drug treatment has human consequences: recurrent parasitaemia, severe malaria, anaemia, and associated morbidity and mortality. In the early 2000s, resistance to single antimalarials led to policy changes recommending deployment of ACTs [3]. In contrast, resistance to the most widely used class of insecticide, pyrethroids, was first documented in the 1980s, but pyrethroid monotherapies still dominate current control efforts [4].

This paper aims to review developments in drug and insecticide resistance over the past 5 years (Box 1), discuss gaps in knowledge, and identify key research priorities (Box 2).

**Box 1. Progress over the past 5 years in drug and insecticide resistance research**

- A promising pipeline of new therapeutics, insecticides, and noninsecticidal vector control tools is in development, largely due to the work of the Medicines for Malaria Venture (MMV) and the Innovative Vector Control Consortium (IVCC)
- Recognition of the impact and importance of drug and insecticide resistance with the creation of the WHO Global Plan for Insecticide Resistance Management in malaria vectors (GPIRM) and WHO Global Plan for Artemisinin Resistance Containment (GPARC)
- Identification of genes and molecular markers associated with drug and insecticide resistance
- Improved understanding of resistance mechanisms in parasite and vector populations
- Global databases to monitor drug and insecticide resistance
- Development of new tools to study resistance in vivo and in vitro, e.g., ring-stage survival assay, parasite clearance estimator, human blood-stage challenge studies for drug resistance, and bioassays that measure the intensity of insecticide resistance

**Box 2. Research and development agenda for drug and insecticide resistance**

**Crosscutting issues for drug and insecticide resistance**

**Applied research**.

- Use in vitro, in vivo, and mathematical models to identify new combinations of drugs and insecticides, and understand how mechanisms of action and mechanisms of resistance inform this
- Determine which conditions are optimal for the emergence and spread of drug and insecticide resistance and how these can be minimised
- Evaluate whether resistance management strategies can restore susceptibility to drugs and insecticides
- Evaluate how new intervention types/paradigms should be introduced and assessed to limit the selection of resistant phenotypes
- Evaluate the optimal surveillance systems for resistance and determine the appropriate data that must be collected (including technical approach, frequency, geography, and temporal–spatial factors)
- Determine and validate the relationships between molecular markers and parasite/vector resistance phenotypes in different transmission settings

**Policy and advocacy**.

- Develop a framework to cost-elimination strategies that accounts for resistance management practices and increasing cost per case of malaria/malaria death averted and identify sources of funding for these strategies
- Agree on the process and minimum data required by the normative bodies to enable a new drug or insecticide product to complete the route to market
- Devise market strategies and incentives to ensure a mix of drug and insecticide products remains available and is used strategically to manage resistance
- Assess which decision-support systems can efficiently and rationally be adapted to drug and insecticide policies
- Determine the minimum dataset required to guide drug and insecticide resistance management and the level of evidence required to switch to new drug or insecticide strategies

**Insecticide resistance**

- Analyse the most cost-effective ways of slowing the spread and emergence of insecticide resistance (e.g., by using a combination of interventions, spatial mosaics, or mixtures of insecticides)
- Determine which spatial and temporal scale insecticide resistance management strategies should be carried out
- Study how much insecticide resistance has a negative impact on mosquito fitness survival or parasite development in the mosquito and investigate how this compares for different active ingredients
- Develop a method to assess the age of resistant mosquitoes
- Define the optimal use of bioassays and molecular markers to accurately predict the efficacy of vector control in relation to insecticide resistance
- Study the mechanisms of mosquito behavioural resistance and assess if this is sustained across generations
- Assess which novel, noninsecticidal tools for controlling mosquito populations would help to slow or prevent the emergence of resistance or restore susceptibility

**Drug resistance**

- Evaluate if the timing of community-based prevention, e.g., mass drug administration, can be optimised to reduce the risk of emerging drug resistance
- Investigate why drugs such as quinine are less likely to develop resistance and use this knowledge for future drug development
- Determine which approaches are most sensitive and specific to determine true drug treatment efficacy (e.g., molecular correction) in *P*. *falciparum* and *P*. *vivax* parasites
- Define what studies are required by policy makers to evaluate the use of multiple therapies
- Define the minimal criteria for inclusion of existing and new drugs in multiple agent regimes (e.g., efficacy, resistance, pharmacokinetic factors, and drug–drug interactions) and whether these criteria change in different programmatic modes
- Study the extent to which human immunity masks the presence of drug resistance, especially resistance to artemisinins

## Methods

The findings presented in this paper result from an extensive literature review of published and unpublished materials and the deliberations of the 2015 malERA Refresh Consultative Panel on Insecticide and Drug Resistance. Electronic databases were systematically searched for published literature between January 1, 2010, and November 2, 2015, without language limitations. Panellists were invited to recommend additional literature. A 2-day workshop was held with the majority of the panel members, including specialists from basic science and product development, field researchers, and WHO representatives. The panel broke into 2 working groups to identify the problems that need to be solved in insecticide and drug resistance and what research is needed to address these problems. Each group fed back to the plenary session, in which further robust discussions and input occurred. This helped refine the opportunities and gap areas in which research is needed. The final findings were arrived at with input from all panellists and several iterations of the manuscript.

### What do we know about resistance?

Insecticides for malaria vector control are limited to pyrethroids for long-lasting insecticidal nets (LLINs) and pyrethroids, organochlorines, organophosphates, and carbamates for indoor residual spraying (IRS). Vector resistance has been detected across Africa to all insecticide classes. However, resistance to the pyrethroids is the most widespread [5]. In Asia, insecticide resistance is common in some *Anopheles* species [6]. Sixty countries have reported resistance to at least 1 insecticide, but the scale of the problem is likely to be much greater [7].

Despite ubiquitous pyrethroid resistance in some areas, millions of pyrethroid-impregnated nets are distributed annually. Once distributed, these nets can contribute to the selection of resistant vectors for the duration of their 3-year life. In Burkina Faso, the intensity of the pyrethroid resistance seen in *A*. *gambiae* increased 10-fold in a single year [8], and this trend is apparent in multiple locations throughout Africa [9]. *A*. *funestus* also exhibits resistance to multiple insecticides at increasing intensities [10–12]. Proactive defensive strategies are critical to reducing the spread and emergence of resistant phenotypes and preventing broad-spectrum cross resistance to multiple insecticides.

In the case of the antimalarials chloroquine and sulfadoxine-pyrimethamine (SP), resistant *P*. *falciparum* and *P*. *vivax* parasites evolved in the Greater Mekong Subregion (GMS) and the island of Papua and South America, respectively [13,14]. Retrospective analysis of molecular markers showed resistant *P*. *falciparum* parasites spread from Southeast Asia foci across Asia and throughout Africa over several decades [15–18]. ACTs were promoted to prevent or retard the selection of resistance by simultaneously administering 2 drug components with different modes of action [19]. However, resistance to artemisinins and their partner drugs is spreading and emerging independently among *P*. *falciparum* populations in the GMS [20–23].

### Identifying resistance

Two main mechanisms of insecticide resistance have been identified: target site mutations (such as *kdr* and *ace)* [24,25] and metabolic resistance involving mutation, duplication, or altered regulation of enzymes and transporters that increase insecticide metabolism or excretion. Metabolic resistance has greater implications for malaria vector control because the efficacy of a range of insecticides is usually affected [5,26].

Routine monitoring of insecticide susceptibility uses phenotypic bioassays that expose live mosquitoes to a single dose of a given insecticide over a fixed time period and measure mortality. The results are highly variable; hence, more laborious methods utilising a range of insecticide concentrations may be needed [27]. These assays have local utility but are often logistically challenging. Larger numbers of mosquitoes can be screened using molecular techniques, although it is unclear under what conditions validated molecular markers could serve as a replacement for phenotypic assays or if this might be appropriate for malaria control programmes [28].

The mechanisms of insecticide resistance can manifest as major changes in the insect nervous system or metabolome. Resistance may have an effect on insect longevity, mating competitiveness, and vectorial capacity [29,30]. Alongside physiological resistance, there is potentially also behavioural resistance, as increased mosquito numbers that bite or rest outdoors have been observed. There is limited evidence on the genetic basis of behavioural resistance, but determining whether vector control interventions are selecting a heritable trait warrants further research [31].

Resistance to artemisinins is assessed in clinical studies by measuring the parasite clearance in a patient in the first several days after treatment [32]. A lab-based assay that correlates with the in vivo parasite response to artemisinins has also been validated [33]. Mutations in the propeller domain of Kelch 13 (PF3D7_1343700) (K13) were identified as a major determinant of artemisinin resistance and may be reliable molecular markers in the GMS [34,35]. Outside the GMS, parasites with K13 mutant alleles are present in many areas at low levels; there is currently no molecular evidence to suggest that these alleles are being selected [22,36–38]. More than 100 K13 mutant alleles have been reported outside of Southeast Asia [22,38–40], but none have yet been associated with the slow-clearing phenotype [41]. One hypothesis is that artemisinin resistance may require additional genetic determinants in these locations to allow selection of K13 mutant parasites that exhibit the slow-clearing phenotype in vivo [20,42]. Nevertheless, the adoption of molecular markers to monitor drug resistance has been much faster than markers to assess insecticide resistance.

Molecular markers correlated with resistance to nonartemisinin antimalarials have also been identified. Polymorphisms or multicopy numbers in the *P*. *falciparum* chloroquine resistance transporter (*pfcrt*) and *P*. *falciparum* multidrug resistance 1 (*pfmdr1*) genes have been associated with resistance to chloroquine and mefloquine [43,44] and polymorphisms or multicopy numbers in the *P*. *falciparum* dihydrofolate reductase (*pfdhfr*) and *P*. *falciparum* dihydropteroate synthase (*pfdhps*) genes have been associated with resistance to SP [45]. Changes in the prevalence of *pfcrt* and *pfmdr1* alleles have been observed in many areas where ACTs including amodiaquine or lumefantrine have been intensively used [46,47]. However, clinical efficacy of leading ACTs that include lumefantrine, amodiaquine, piperaquine, or mefloquine appears to remain acceptable in areas outside the GMS. Recent research suggests that plasmepsin 2–3 is associated with clinical and in vitro piperaquine resistance (PSA, piperaquine survival assay) but other markers could also be involved [48]. In Southeast Asia, intensive use of dihydroartemisinin-piperaquine (DP) in parasites already resistant to piperaquine and artemisinin has selected parasites with multiple resistance mechanisms, and high levels of treatment failure to DP are now observed in Cambodia [49].

Chloroquine remains the recommended treatment for *P*. *vivax*, but resistance and declining efficacy has been noted in several populations, and ACTs are recommended in some areas [50,51]. There are no standardized molecular correlates of chloroquine resistance for *P*. *vivax*, but *P*. *vivax* multidrug resistance 1 (*pvmdr1*) has been associated with resistance [52]. Beyond this, the understanding of resistance in nonfalciparum malaria is very limited.

### Public health impact of resistance

While ecological studies have found broad evidence of dramatic health effects of spreading drug resistance [18], efficient assessment of the public health impact of antimalarial and insecticide resistance has been difficult. First, assessments of resistance prevalence are drawn from a few sentinel sites, but the heterogeneity of resistance in neighbouring populations can be enormous, making specific predictions difficult. Second, molecular markers are easier to measure at finer spatial and temporal scales, but the relationship with the drug or insecticide response is not direct [53,54]. Third, most policies on malaria treatment and vector control are implemented nationally, so recommending policies for regions within a country may be operationally unfeasible.

Drug resistance increases the risk of treatment failure and therefore transmission, but these relationships can be difficult to establish in the field. Human factors, especially immunity, affect treatment efficacy, so treatment failure in the whole population is not obvious until parasite resistance is well established [55]. However, in children there is a clear relationship between parasitaemia and anaemia, with associated morbidity and mortality [55,56]. Studies have correlated the prevalence of molecular markers with the risk of treatment failure, but no metric that works in all regions has been defined [57]. As a result, the prevalence of molecular markers has had a limited impact on policies for routine antimalarial use [58]. This disconnect is changing in the GMS, where ACT treatment failure has reached crisis levels [59], and rapid assessments of molecular markers for resistance to artemisinins and partner drugs are currently being used [47].

There are few published studies on the epidemiological impact of insecticide resistance, so decisions rely primarily on entomological end points. Evidence from a 5-country evaluation attempted to assess whether LLINs remain effective in the presence of pyrethroid resistance, although the studies were in areas with low to moderate resistance as measured in single-dose bioassays without assessment of resistance intensity [60]. This study was not able to quantify the effect on LLINs [59]. For IRS, the best evidence for an epidemiological impact of pyrethroid resistance comes from settings where pyrethroids were replaced in IRS campaigns with alternative insecticides and parasite prevalence rapidly declined [61, 62]. Similar evidence is available from a study in an area of Sudan with pyrethroid resistance but carbamate susceptibility, in which IRS with pyrethroids in addition to LLINs had no added impact, but changing to carbamate IRS halved the malaria incidence [60].

### Managing resistance, moving toward elimination

#### Optimizing drug and insecticide use

Avoiding parasite or mosquito population exposure to a single selective agent is the central principle of resistance management. Ideally, insecticidal compounds with different modes of action should be used simultaneously or in spatial or temporal rotation. These principles, which are identical to those used in the management of insecticides used for crop pests, have been outlined in the GPIRM in malaria vectors [63]. Unfortunately, implementation has been challenging; pyrethroid resistance is ubiquitous, nonpyrethroid LLINs are not currently available, and other forms of vector control can significantly increase costs [64]. New public health insecticides with different modes of action are on the horizon [65], but we lack information on the effectiveness of the proposed strategies to slow the emergence or spread of insecticide resistance, and there is no clear indication of how they should be integrated alongside existing tools. This includes those that are noninsecticidal and products that work on different targets, e.g., spatial repellents and endocticides, whose efficacy may not be influenced by insecticide resistance [65]. Another confounder is the application of most insecticides for both agricultural and public health use. The impact of this on public health is highly variable depending on crop type and volume and timing of insecticide application.

What are the benefits of insecticide rotations, mixtures, or spatial mosaics of different compounds? What is the impact of adding nonpyrethroid IRS where LLINs are already deployed at high coverage and quality? When should new insecticides be adopted? What is the ideal rotation period or mosaic configuration? How many insecticide classes are needed for effective rotation or mosaic strategies? Despite the absence of data to answer these questions, some countries have already developed operational frameworks for resistance management that could be adopted by other programmes [66].

ACTs are still effective in most regions outside the GMS. Optimisation of dose, duration of treatment, timing of treatment, and pharmacokinetic-dynamic profiles in specific subpopulations, e.g., children and pregnant women, should be systematically encouraged post-licensure to maximise efficacy and slow selection for resistance. Pooled analyses have assessed the effect of dosing strategies for the several currently used ACTs, but the uptake of this by malaria control programmes is limited [67]. Molecular markers are being used in addition to therapeutic efficacy studies in specific locations in the GMS to choose treatment policies more accurately [67], but far more complete information on all ACTs is needed.

Different published models diverged on the conclusion that implementation of multiple first-line therapies could more effectively prevent the emergence of drug resistance compared with the temporal rotation or sequential use of first-line treatments [68–71]. Multiple models need to be evaluated and studies to verify this must be defined [72]. We also need to better understand why parasites do not seem to have developed resistance to quinine and factor this into future drug development efforts. In Southeast Asia, the use of triple therapies using existing antimalarials is currently being tested and could be considered in the context of multidrug-resistant malaria [73].

Assessment of the selective pressures and emergence of resistance to antimalarials is difficult with small-scale studies, but large-scale public health interventions may provide evidence. For example, studies should be undertaken in countries using different drug combinations for treatment and mass chemoprevention campaigns, such as seasonal malaria chemoprevention, mass drug administration, or intermittent preventive treatment in pregnancy (IPTp). Coordination of these interventions in the same locality may provide one way to reduce or disrupt the selection pressure exerted on a single class of compound [74].

#### Using data to support resistance management

Entomological data generated by countries vary in quantity and quality, and limited information flow between entomologists, programme managers, and research institutes has hindered advocacy efforts around improved resistance management. Linking entomological data to epidemiological outcomes is extremely complex [75] and by the time resistance has a demonstrable public health impact, it may be too late to intervene against it. However, South Africa [62], Zambia [76], and Equatorial Guinea [64] have resistance management plans in place. Similarly, molecular marker surveillance can inform which drug regimens are the most suitable for particular programmatic modes. This approach is now routine in some African countries [74,77] but is not universal. Drug-resistance monitoring in some countries also requires strengthening, and despite the tighter link to public health impact, the ability to respond rapidly may be lost if resistance monitoring is not well embedded. For both insecticides and drugs, defining the minimum essential data required for policy makers to manage resistance and ensuring that programs employ rigorous quality assurance in collecting and managing these data are critical.

Resistance surveillance is weak in many endemic countries. Inadequate attention and funding have been allocated to entomological monitoring and insecticide susceptibility research. Several countries in Africa have established sentinel sites for the longitudinal monitoring of insecticide resistance [5]. However, the methods, timing, and sampling are inconsistent, making meaningful inferences difficult [78]. Most of these sites use discriminating dose assays [79]. All bioassays are performed on 3–5-day-old mosquitoes under standard insectary conditions, so the effect of natural mosquito traits (e.g., age, blood-feeding status, circadian rhythm [80,81], and climatic variables [82]) on resistance is not assessed or reported [83]. Molecular species identification of mosquitoes undergoing resistance tests may also increase accuracy when compared to morphological identification. Techniques have been developed to measure the age distribution of mosquito populations in the laboratory [84], but a more precise, low-cost, field-applicable method is needed to allow malaria control programmes to evaluate the efficacy of vector control interventions is needed.

#### Anticipating the challenges of lower transmission

High-level use of interventions can suppress malaria transmission but also increases the risk of selection of resistance, creating new challenges at the later stages of elimination. Resistance surveillance in low transmission regions is increasingly expensive, and maintaining human and material capacity in the context of many other public health needs is crucial. The minimal criteria for the inclusion of new or existing therapeutics or insecticides in a multi-agent regimen must be defined. For drugs, these criteria might depend on transmission levels and could include pharmacokinetic-dynamic profiles, mechanisms of resistance, cross resistance, and drug–drug interactions. The corresponding parameters for insecticides of persistence/residual efficacy, mechanisms of resistance/cross resistance, or compound interactions are equally relevant. If a robust resistant phenotype can be defined, whole genome sequencing of parasites and vectors can identify regions under selection very early in the process, giving clues to associated genetic changes [85].

#### Market strategies and getting products to market

Single first-line antimalarial treatments or insecticide monotherapies may be cheaper in the short term, but the long-term cost-effectiveness will be compromised by increasing levels of resistance [86]. Development of normative guidance on product use within a multiyear programme of interventions is essential if short-term decision-making is to change. The selection of products may be based on a number of epidemiological, entomological, logistic, and financial variables. It is critical to develop a framework that reliably costs the long-term elimination strategies, rather than short-term ‘delivered units’, and takes into account resistance management practices. As we head toward elimination, the increased cost of keeping drugs and insecticides available for a diminishing number of cases means incentives and market strategies for keeping the pipeline of products active are paramount.

Clarity is needed on the evidence required by normative bodies to approve new products and develop treatment guidelines. New tools are likely to have a higher unit price, so clear data requirements and paths to their use require definition. Without this, programme financial constraints; uncertainties around cost-effectiveness; and delays in recommendation, production, and procurement could mean products to overcome resistance are underutilised. If this situation becomes the norm, incentives for innovation will diminish and the pipeline of efficacious tools will soon be depleted.

## Conclusion

Resistance is an inevitable consequence of drug and insecticide treatment, but the malaria community as a whole has repeatedly failed to respond to this issue in a proactive way. Programme and policy decisions should be based on comprehensive resistance data, and this should be coupled with improved efforts to understand the complex biological processes that select for resistant phenotypes. The tools to surmount resistance are limited and little is known about the most effective resistance management measures, so new therapeutics and vector control products should have a clear route to market and be carefully implemented and evaluated to optimise the choice of interventions. Multidrug and insecticide regimens are not unique to malaria control and other disease systems such as HIV [87], tuberculosis [88], and agricultural pest control [89] offer important insights into the management of insecticide- and drug-based approaches. The malaria community must learn from other disease groups and industries and heed the lessons of the past or risk further erosion of the malaria elimination agenda as renewed efforts are undermined by resistance.

## Abbreviations

ACT: artemisinin-based combination therapy
DP: dihydroartemisinin-piperaquine
GMS: Greater Mekong Subregion
GPARC: Global Plan for Artemisinin Resistance Containment
GPIRM: Global Plan for Insecticide Resistance Management
IPTp: intermittent preventive treatment in pregnancy
IRS: indoor residual spraying
IVCC: Innovative Vector Control Consortium
K13: Kelch 13 (PF3D7_1343700)
LLIN: long-lasting insecticidal net
malERA: Malaria Eradication Research Agenda
MESA: Malaria Eradication Scientific Alliance
MMV: Medicines for Malaria Venture
pfcrt: *P*. *falciparum* chloroquine resistance transporter
pfdhfr: *P*. *falciparum* dihydrofolate reductase
pfdhps: *P*. *falciparum* dihydropteroate synthase
pfmdr1: *P*. *falciparum* multidrug resistance 1
PSA: piperaquine survival assay
SP: sulfadoxine-pyrimethamine
pvmdr1: *P*. *vivax* multidrug resistance 1
